# Identification of signaling components required for the prediction of cytokine release in RAW 264.7 macrophages

**DOI:** 10.1186/gb-2006-7-2-r11

**Published:** 2006-02-20

**Authors:** Sylvain Pradervand, Mano R Maurya, Shankar Subramaniam

**Affiliations:** 1 Bioinformatics and Data Coordination Laboratory, Alliance for Cellular Signaling, San Diego Supercomputer Center, University of California at San Diego, Gilman Drive, La Jolla, CA 92093, USA; 2 Department of Bioengineering, University of California at San Diego, Gilman Drive, La Jolla, CA 92093, USA; 3 Department of Chemistry and Biochemistry, University of California at San Diego, Gilman Drive, La Jolla, CA 92093, USA

## Abstract

**Background:**

Release of immuno-regulatory cytokines and chemokines during inflammatory response is mediated by a complex signaling network. Multiple stimuli produce different signals that generate different cytokine responses. Current knowledge does not provide a complete picture of these signaling pathways. However, using specific markers of signaling pathways, such as signaling proteins, it is possible to develop a 'coarse-grained network' map that can help understand common regulatory modules for various cytokine responses and help differentiate between the causes of their release.

**Results:**

Using a systematic profiling of signaling responses and cytokine release in RAW 264.7 macrophages made available by the Alliance for Cellular Signaling, an analysis strategy is presented that integrates principal component regression and exhaustive search-based model reduction to identify required signaling factors necessary and sufficient to predict the release of seven cytokines (G-CSF, IL-1α, IL-6, IL-10, MIP-1α, RANTES, and TNFα) in response to selected ligands. This study provides a model-based quantitative estimate of cytokine release and identifies ten signaling components involved in cytokine production. The models identified capture many of the known signaling pathways involved in cytokine release and predict potentially important novel signaling components, like p38 MAPK for G-CSF release, IFNγ- and IL-4-specific pathways for IL-1a release, and an M-CSF-specific pathway for TNFα release.

**Conclusion:**

Using an integrative approach, we have identified the pathways responsible for the differential regulation of cytokine release in RAW 264.7 macrophages. Our results demonstrate the power of using heterogeneous cellular data to qualitatively and quantitatively map intermediate cellular phenotypes.

## Abstract 2

An integrative approach is used to identifying the pathways responsible for the release of seven cytokines in response to selected ligands.

## Background

A main component of the inflammatory response is the production and release of immuno-regulatory cytokines and chemokines by macrophages. Pro-inflammatory cytokines, such as tumor necrosis factor (TNF)α, interleukin (IL)-1, IL-6, IL-12, granulocyte macrophage colony stimulating factor (GM-CSF) and interferon (IFN)γ, induce both acute and chronic inflammatory responses; the chemokines MIP(macrophage inflammatory protein)-1α and RANTES (Regulated on Activation, Normal T Expressed and Secreted) are involved in the chemotaxis of leucocytes; and anti-inflammatory cytokines, such as IL-4, IL-10 and transforming growth factor (TGF)β, limit the magnitude and the extent of inflammation [1,2]. Activated macrophages synthesize and secrete cytokines [3]. This process is mainly regulated transcriptionally, although post-transcriptional and translational mechanisms may also play a role [4,5]. Several pathways transmit the signals that trigger cytokine production. Among them, the nuclear factor kappa B (NF-κB) pathway plays an essential role in activating genes encoding cytokines [6]. Other signaling pathways, such as mitogen-activated protein kinases (MAPK), signal transducer and activator of transcription (STAT), cAMP-protein kinase A (PKA), interferon regulatory factor (IRF) or CAAT/enhancer-binding proteins (C/EBP), have also been described to be invoked in macrophages [1,7]. These pathways are not distinct entities, but are part of a general network whose different signals are produced by multiple stimuli that generate different cytokine responses.

Systems Biology approaches to cellular networks are based on integration of diverse read-outs from cells. The contextual dependence of the pathways on the cell state and its response to specific inputs renders our ability to understand every network in entire detail a near impossibility. However, quantitative mapping of the input to response of a given phenotype often can be achieved in a more coarse-grained manner with appropriate analyses of the read-outs. This is our leitmotif in this work. Such an approach allows the elucidation of the common and different signaling modules required for the release of different cytokines, and the quantitative prediction of amounts of cytokines released.

**Figure 1:**
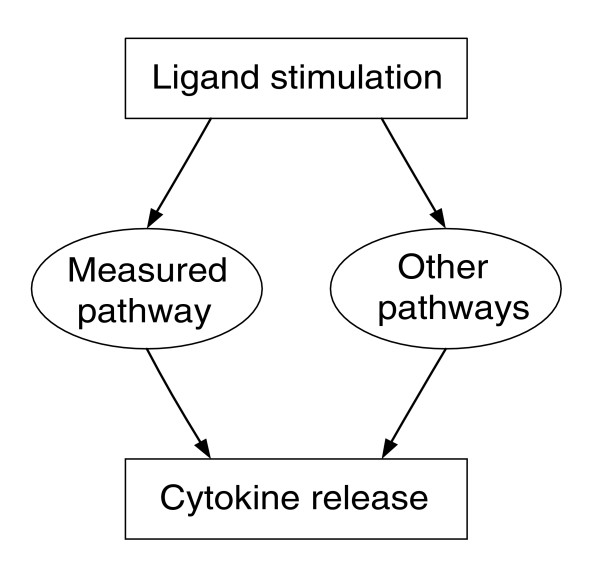
Schematic representation of the experimental data. RAW 264.7 macrophages were stimulated with different combinations of ligands. Signals leading to cytokine release were transmitted not only through the 22 signaling proteins and a second messenger that were recorded (measured pathways), but also through other pathways (unmeasured pathways).

The Alliance for Cellular Signaling (AfCS) [8,9] has recently generated a systematic profiling of signaling responses in RAW 264.7, a macrophage-like cell line (AfCS data center [9]). From this dataset, an input-output model is generated in which signaling responses (input) are used to predict cytokine release (output) (Figure 1). Since all signaling pathway activations are not measured (for example, STAT6), our model includes an alternative branch going directly from the stimulus to the response that accounts for ligand-specific unmeasured pathways. Here, we propose a novel integrated approach that uses principal-component-regression (PCR) and a model-reduction procedure to develop necessary and sufficient models that predict cytokine release based on signaling pathway activation [10]. Given that these minimal models contain only the essential components, the number of signaling predictors not biologically involved in cytokine release (false positives) is reduced considerably. We show that this data-driven approach is able to capture most of the known signaling pathways involved in cytokine release and is able to predict potentially important novel signaling components. This strategy allows classification of cytokine responses based on the activation of their signaling modules and predicts an estimate of the amount of cytokine released.

## Results

### Signaling pathways and cytokine release after ligand stimulation

**Figure 2:**
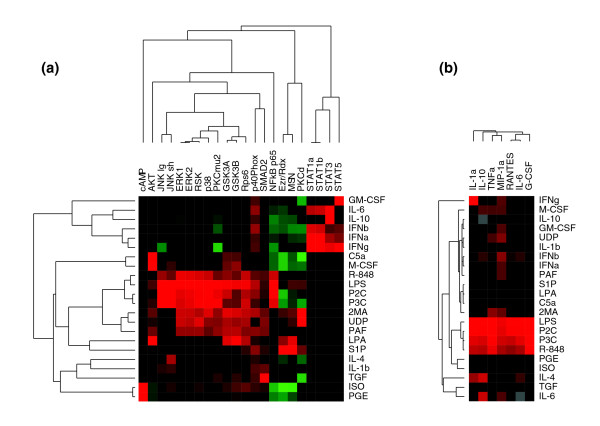
Two-way hierarchical clustering of the RAW 264.7 macrophage. **(a) **Signaling pathway responses and **(b) **cytokine release after single ligand stimulations. Average linkage clustering was performed using un-centered Pearson's correlation metrics on log-transformed and variance-normalized data. Data are averages over the different time points and across repeated experiments. Red = positive change; green = negative change.

The AfCS provides a global profiling of signaling responses and cytokine release to a set of 22 ligands applied alone or in combinations of two (AfCS data center [9]). Global-response patterns to single-ligand stimulations were first visualized using two-way hierarchical clustering (Figure 2a, b). Clustering of activated signaling proteins (studied through phosphoprotein measurements) and cAMP production after ligand stimulation showed a consistent classification of ligands along their known families (Figure 2a). We observed a cluster of STAT activator cytokines (GM-CSF, IL-6, IL-10, IFNα, IFNβ and IFNγ), a cluster of Toll-like receptor-activating ligands (R-848, LPS, PAM 2 and PAM 3), a cluster of G protein α_q_-activating ligands (2MA, PAF, UDP), which strongly activate ERK1/2 and p38 but not JNKs, a cluster of G protein α_s_-activating ligands (ISO and PGE), and a cluster of lysophospholipid agonists (LPA, S1P). IL-1β, which did not show any strong response, and IL-4, whose main signaling target (STAT6) was not measured, clustered together as weak inducers. Although not directly related, G protein α_i_-activating ligand C5a and tyrosine kinase receptor ligand M-CSF were classified together for their strong activation of Akt. In hierarchical clustering of signaling responses, a strong correlation was observed between ERK1/2 activation and the activation of their downstream target RSK, as well as between ERK1/2 activation and p38 activation. Clustering of the cytokine release data showed an overall similar pattern for all cytokines released, with a strong response to Toll-like receptor (TLR) ligands and a weaker or no response to other ligands (Figure 2b). The release of a few cytokines were strongly affected by some ligands; for example, IL-1α by IFNγ and IL-4, and IL-10 by IL-4 and IL-6. These clustering analyses gave a first insight into the connectivity between signaling pathway activation and cytokine release by looking at responses triggered by the same set of ligands. For example, a strong connectivity can be derived between phosphoproteins JNKs and NF-κB p65 and all cytokines from the fact that TLR ligands strongly activate all of them.

### Correlations between signaling pathway activation and cytokine release

**Figure 3:**
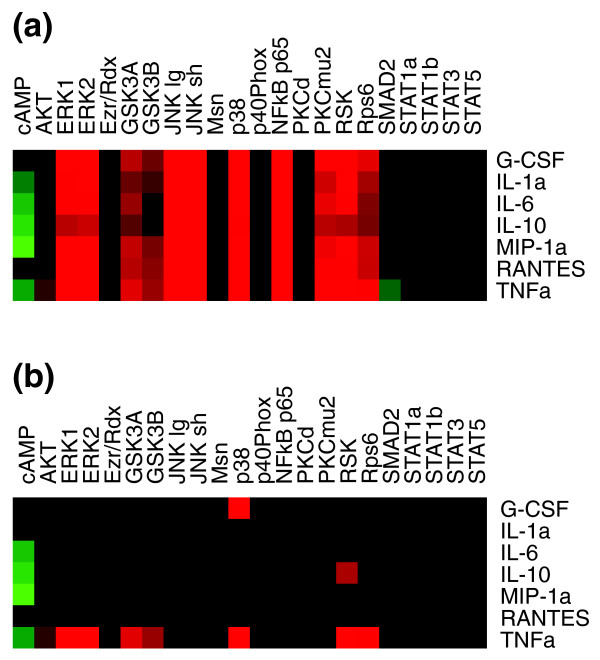
Correlation coefficients between signaling responses and cytokine release. Pearson's correlation coefficients were computed for each pair of signaling responses and cytokines using data from single- and double-ligand stimulations. Data from TLR ligand stimulation were **(a) **included in the procedure or **(b) **excluded from the procedure. Data were log-transformed and variance-normalized. Significance of correlations was assessed following a *t *distribution. Heat maps were produced from significant correlation coefficients (red = positive correlation; green = negative correlation).

To further investigate the association between signaling pathway responses and cytokine release, correlation coefficients were calculated based on data from single- and double-ligand screens. As shown in Figure 3a, the overall patterns of correlation were similar for different cytokine releases. Indeed, significant positive correlations were observed between activation of any of ERK1/2, GSK3A, GSK3B, JNKs, p38, NF-κB, PKCμ2, RSK or Rps6 and any of the cytokine releases (except between GSK3B and IL-10/IL-1α). The only remaining significant positive correlation was between Akt phosphorylation and TNFα release. Significant negative correlations were observed between production of the second messenger cAMP and all cytokine releases except GCSF and RANTES, as well as between SMAD2 phosphorylation and TNFα release. Since TLR ligands strongly activate most of the signaling pathways, correlations were computed after omission of TLR ligand data in order to uncover potentially important features (Figure 3b). Without TLR ligand data, only a few positive correlations were observed, most of them involving TNFα. The phosphorylation of STAT proteins showed weak correlations with IL-1α, IL-10, MIP-1α and RANTES responses that were not significant when TLR ligand data were included. All significant negative correlations between cAMP production and the different cytokines released were conserved except for release of IL-1α. These correlation coefficients suggest direct connections between signaling proteins and cytokines. However, simple correlation coefficients do not take into account the high correlations among signaling proteins themselves and include a large number of non-causal relationships.

### Identification of cytokine regulatory signals among measured signaling pathways

**Figure 4:**
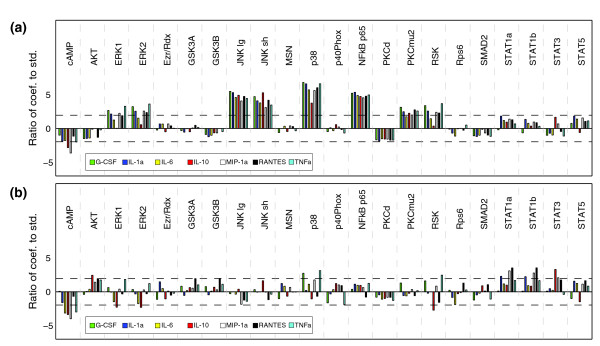
Significance of signaling-pathway predictors for cytokine release. Data from TLR ligand stimulation were **(a) **included or **(b) **excluded. PCR analyses were performed as described in Materials and methods. For a given output, significance of signaling responses was measured as the ratio of their regression coefficients (coef.) divided by the standard deviation (std) of coefficients corresponding to random outputs from the same population as the actual outputs (see Materials and methods). Averaged ratios outside a 95% confidence interval (horizontal dashed lines) are considered significant.

In order to define the contributions of each signaling component to cytokine release, PCR models were developed. PCR was chosen as the method for analysis because it takes into account correlations among predictors (that is, signaling pathway activation) and reduces the dimension of the data set in order to define a linear model that predicts the responses (that is, cytokine release). PCR and related modeling techniques have been shown to be appropriate choices for analyses of biological data that are highly variable in nature [11]. Figure 4 displays the significance of the regression coefficients for the 22 signaling pathway predictors with (Figure 4a) and without (Figure 4b) TLR ligand data. As expected, strong similarities are observed between correlation coefficients and significant PCR regression coefficients. When TLR ligands were included, the strongest overall regression coefficients were for the two JNK isoforms, p38 and NF-κB p65. PKCμ2 was less prominent, but was still significant for all except IL-6. ERK1, ERK2 and RSK shared a similar profile and were all significant for G-CSF, IL-1α, MIP-1α, RANTES and TNFα. Most of these coefficients lost their strength when data from TLR ligands were removed (Figure 4b). The remaining positive coefficients were p38 for G-CSF and TNFα and RSK for TNFα. As for correlation coefficients, STAT proteins became significant for releases of IL-1α (STAT1α/β), IL-10 (STAT3), MIP-1α (STAT1α/β and 3) and RANTES (STAT1α/β). In both datasets, cAMP had a significant negative coefficient for IL-10, MIP-1α, TNFα and IL-6 (the lastonly when without TLR ligand data). This PCR analysis captured cytokine release associated with signaling pathways for which measurements are available. However, it is well established that other pathways (for example, STAT6, IRFs, C/EBPβ) are important in cytokine synthesis and release.

### Analysis of the residuals to identify significant ligands

**Figure 5:**
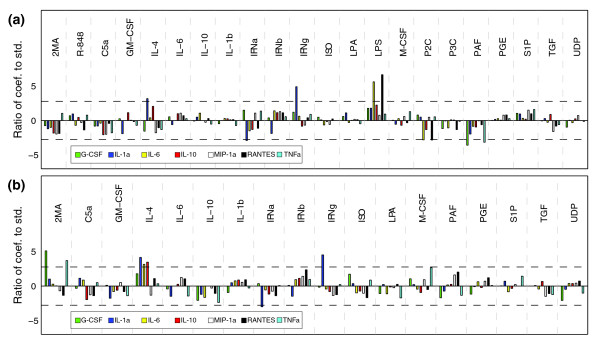
Significance of ligand predictors for cytokine release residuals. Data from TLR ligand stimulation were **(a) **included or **(b) **excluded. Residuals of cytokine release measurements were calculated from PCR models using signaling pathways as predictors. PCR analyses were performed on the residuals as described in Materials and methods. Averaged ratios outside a 95% confidence interval after noise correction (horizontal dashed lines) are considered significant. Since these residuals also carry noise, we applied a corrective factor to set a higher confidence interval to identify significant ligands (see Materials and methods).

In order to take into account the participation of pathways not associated with measurements, we repeated PCR analysis on the part of the cytokine responses that was not fitted by the measured activated signaling pathways (that is, residuals). In this instance, we used the ligands as predictors to fit the residual. Few correlations emerged among regression coefficients of the ligands and only a few ligands were statistically significant (Figure 5a, b). The significant positive coefficients were: IL-4 for IL-1α, IL-6 and IL-10 releases (in the case of IL-6 and IL-10, only when TLR-ligand data was not used); IFNγ for IL-1α release; LPS for IL-6 and RANTES releases; as well as 2MA for G-CSF and TNFα releases in non-TLR ligand data (Figure 5a, b). Significant negative coefficients seemed to be compensatory. Indeed, IFNγ strongly activated both STAT1α/β phosphorylation and IL-1α release, whereas IFNα strongly activated STAT1α/β phosphorylation, but did not activate IL-1α release (Figure 2). Since part of the effect of IFNγ on IL-1α was captured by the positive regression coefficients of STAT1α and β, this might be compensated in the residuals through a negative coefficient for IFNα. Similar arguments can be applied for the negative coefficients of P2C for IL-6 and RANTES releases. Indeed, regression coefficients of the different measured pathways activated by TLR ligand may have been overestimated in trying to fit the specific LPS effect. The negative coefficients of PAF for G-CSF and TNFα releases (TLR ligand data) should be evaluated along with the positive coefficients of 2MA (non-TLR ligand data). Indeed, both ligands are strong activators of ERK1/2 and p38. With TLR ligand data, these two signaling pathways had large regression coefficients that captured G-CSF and TNFα responses after 2MA stimulation accurately, but overestimated them after PAF stimulation. Without TLR ligand data, regression coefficients of ERK1/2 and p38 were smaller and not sufficient to capture the response after 2MA stimulation. A final related observation was that the overall patterns of regression coefficients for G-CSF and TNFα release were highly similar and may reveal a common regulatory mechanism.

### Minimal models of cytokine release

**Figure 6:**
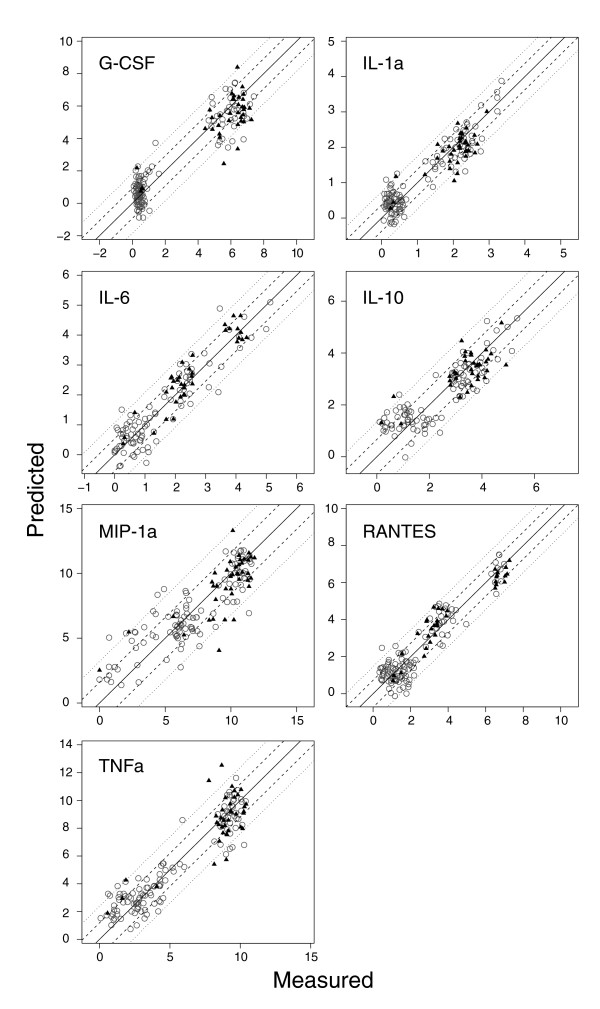
Prediction of training and test data on cytokine release using PCR minimal models. Measured versus predicted log-transformed concentration values are indicated for training data (unfilled circles) and test data (filled triangles). Dashed and dotted lines indicate one and two standard deviations, respectively, from the average predicted fit of the training data.

In the above PCR models, a predictor might be declared significant only because of its high correlation with other important predictors. In order to identify the required signaling pathways and ligands for the cytokine responses, we developed a minimal PCR model. Before model reduction, it was confirmed that PCR models based only on the significant predictors were able to fit the data as well as models based on all predictors (data not shown). Then we identified the smallest set of predictors able to fit the data statistically as compared to a detailed model consisting of all 22 signaling-proteins and 22 ligands (see Materials and methods). This procedure was performed with and without TLR ligand data. The two sets of predictors in the models based on data including or excluding TLR ligands were then combined to produce a single minimal model. All possible combinations of predictors in this single minimal model were tested and the model corresponding to absolute minimal fit error over training data was retained (Table 1). Several regulatory modules were immediately evident from these minimal models. The first module consisted of NF-κB p65 and one of the JNK isoforms and translated the common dependency to TLR ligands for all cytokine releases (except MIP-1α, which did not retain NF-κB p65). The second module included p38 and PAF (as a negative ligand predictor) and underlined a common regulatory mechanism for three different cytokines (G-CSF, MIP-1α and TNFα). The third module is defined by STAT1 transcription factors and is required for the prediction of the release of MIP-1α and RANTES. The last module involving measured signaling activity is inhibitory and is defined by cAMP. IFNγ, IL-4 and LPS were all required for the prediction of more than one cytokine release and each of them may reflect other important regulatory modules. Finally, some ligands were specific in predicting the release of one cytokine (IFNβ for IL-6, IL-6 for IL-10 and M-CSF for TNFα). Figure 6 displays the fits of these different minimal models for training and test data. Most of the training and test data points were inside two root-mean-squared errors of the training data. In the case of MIP-1α, predictors did not yield a good fit. After inclusion of NF-κB p65, an obvious false negative predictor [12], the fit-error improved only slightly (from 2.57 to 2.53 on the training data and from 2.88 to 2.49 on the test data). MIP-1α data are characterized by a high variance and data can simply be difficult to fit because of imprecision in the measurements. G-CSF and TNFα have corresponding outlier points. All over-predicted points involved 2MA stimulation and might be due to an overweighting of the role of p38. The under-predicted points carried an especially low value for the JNK large isoform, NF-κB p65 or p38 and, therefore, may be considered as outliers.

### Network reconstruction

**Figure 7:**
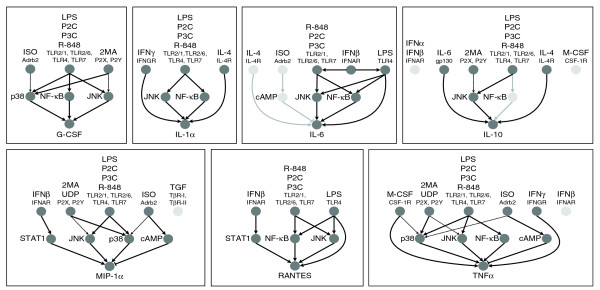
Topologies of signaling networks leading to cytokine releases derived from PCR minimal models and ANOVA analysis. In each panel, nodes in the upper row represent ligands that significantly regulate respective cytokines (ANOVA). Nodes in the middle row represent significant pathways identified by PCR minimal models. Edges between top and middle rows represent significant signaling pathway regulation by the given ligands (ANOVA). Edges between top and bottom rows, or middle and bottom rows, represent significant participation identified by PCR minimal models. Weak activation of signaling pathways is indicated by dashed edges. Light gray: pathways demonstrated in the literature to not play any role (false positives).

In order to develop a coarse-grained network of cytokine production, 152 independent analyses of variance (ANOVA; 7 cytokines times 22 ligands minus 2 cytokines that are also ligands) that identified ligands that significantly enhance cytokine release and 462 independent ANOVA (21 phosphoproteins times 22 ligands) that identified ligands that significantly enhance signaling-protein phosphorylations were considered. The case of cAMP is treated independently and only two ligands (isoproterenol and prostaglandin E2) significantly stimulate its production. To declare a ligand-cytokine or ligand-phosphoprotein link significant, two criteria were used: a *P* value cutoff of 0.05 after correction for multiple testing (Dunn-Sidak); and an absolute change outside a 90% confidence interval of all the basal values for the particular measurements. Connections were then drawn from the ligands that significantly stimulate cytokines to the signaling pathway identified in the PCR minimal models according to activations identified by ANOVA (Figure 7). Ligands from the PCR minimal model that were not consistently identified by ANOVA after single ligand stimulation were investigated for interaction effects using a distinct ANOVA model. IFNβ was shown to have a significant positive interaction with all four TLR ligands on IL-6 release. These networks are compared with known activations from the literature in the discussion.

## Discussion

**Figure 8:**
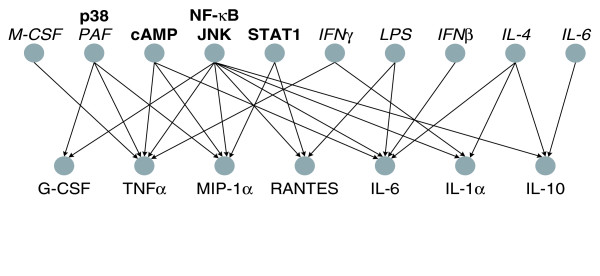
Combined network of signaling components required for the production of cytokines. Upper row represents the different signaling pathway components. Lower row represents the different cytokines. Bold face: signaling component identified from measured signaling pathways. Italic face: signaling component identified from residuals and representing ligand-specific unmeasured pathways.

Cytokines and chemokines released by activated macrophages modulate the inflammatory response [3]. Thus, understanding the regulation of the expression and release of these mediators is crucial for understanding the course of the inflammation process. Here we propose models that derive the responses of seven cytokines from the activation of signaling pathways. These models reasonably predicted cytokine release and identified a total of ten signaling components involved in cytokine release (Figure 8). Four components were defined by measured signaling pathways and six components were defined by ligand-specific signaling pathways. Among them, a NF-κB p65-JNK component was required for the prediction of all cytokine releases and reflected the dependency on TLR ligand inputs. A TLR4 specific component (identified by LPS ligand) was required for the prediction of RANTES and IL-6. The other components reflected TLR ligand independent pathways. Regulation of cytokine expression has been studied extensively (Table 2). Therefore, for each cytokine, information available from the literature was used to evaluate and validate our models.

### G-CSF

G-CSF specifically regulates the production of neutrophilic G granulocytes and enhances the functional activities of mature neutrophils [13]. The expression of the gene encoding G-CSF is regulated by a combination of transcriptional and post-transcriptional mechanisms [14]. Three conserved upstream regions have been identified in the G-CSF promoter, including binding sites for OCT (octamer), NF-κB and C/EBPβ. The last two have been shown to be required for the induction of the gene [13,15]. Our model identified NF-κB, JNK and p38 pathways (Figure 8). C/EBPβ activation was not measured in our experimental data. However, its role may be inferred by the presence of JNK. Indeed, JNK was proposed to contribute to the transcriptional activation of C/EBPβ in macrophages [16]. The presence of p38 in our minimal model may be related to post-transcriptional regulation. It has been shown that G-CSF mRNA contains AU-rich destabilizing elements (AREs) in the 3'-untranslated region [17] and recent evidence suggests a role for the p38 pathway in regulation of ARE mRNA stability [18].

### IL-1α

IL-1α is a pro-inflammatory mediator distinct from IL-1β that is produced by monocytes after various stimulation [19]. In contrast to IL-1β, few studies have investigated the mechanisms that mediate expression of the gene encoding IL-1α [20]. Among transcription factors, AP-1 (a JNK target), NF-κB and Sp1 were shown to regulate expression of this gene [21-23]. In our model, these known activators are reflected through JNK and NF-κB (Figure 8). We also identified IFNγ and IL-4 as potential novel activators through independent pathways.

### IL-6

IL-6 is a pleiotropic cytokine whose expression is mediated by a wide range of signaling pathways that may vary depending on the cell type [24]. In monocytes, a NF-κB site is crucial for LPS-induced expression of the gene encoding IL-6 [25]. In these cells, it has also been shown that a synergistic induction by IFNγ and TNFα involves cooperation between IRF-1 and NF-κB p65 homodimers [26]. IRF-1 is also a down-stream target of IFNβ [27] and has been designated as an immediate-early LPS-inducible gene [28]. In order to activate IRF-1, LPS acts through a MyD88-independent pathway not shared by other TLR ligands [29]. Therefore, in our model, IRF-1 may be represented both as the LPS- and as the IFNβ-specific pathway. The other important non-constitutive transcription factors involved in IL-6 gene activation include AP-1, C/EBPβ, which work synergistically with NF-κB and may be captured by the JNK component of our minimal model [30]. IL-4 and cAMP are the remaining two components of our model (Figure 8). Using ANOVA analysis, we did not see any significant induction of IL-6 production by IL-4; neither did we see any interactive effect of IL-4 with other ligands. IL-4 is known for its inhibitory effects on pro-inflammatory cytokines, although it has been shown to stimulate IL-6 in osteoblast-like cells [31]. Therefore, we may not give a high confidence to an effect of an IL-4 specific pathway on IL-6 cytokine release. A similar problem is observed with cAMP, which was identified as a negative predictor. Several reports have indicated activation of the IL-6 gene by cAMP in monocytes [25], although other reports have shown no response [32]. In our PCR analysis, a lack of response may be translated to an anti-correlated predictor. Since the ligands that lead to elevated levels of cAMP did not decrease IL-6 production, the negative sign of cAMP may not reflect an inhibitory action.

### IL-10

IL-10 is a pleiotropic cytokine that has dominant suppressive effects on the production of pro-inflammatory cytokines by monocytes [33]. Promoter analysis in RAW 264.7 macrophages stimulated by LPS showed a central role for a Sp1 binding site in the activation of the gene encoding IL-10 [34]. On the other hand, this study and others suggest no contribution for NF-κB [35]. The activation of the IL-10 gene by Sp1 was later suggested to be p38 dependant [36]. In addition to Sp1, C/EBPβ and δ factors are also involved in LPS-induced gene expression of IL-10 [37]. Thus, contrary to the other cytokines, TLR ligand pathways that activate IL-10 are p38-Sp1 and C/EBP dependent. Our model only partially reflects these facts through the presence of JNK (Figure 8). Another missing predictor is cAMP, since it is known to elevate IL-10 production [38]. Two ligands (IL-4 and IL-6) were found to have specific pathways that activate IL-10 release. The effects of IL-4 on IL-10 production in macrophages have been contradictory [39]. Indeed, IL-4 suppresses LPS-induced IL-10 production by peripheral blood mononuclear cells, but increases LPS-induced IL-10 production by monocyte-derived macrophages. Stimulation of IL-10 by IL-6 has been reported [40]. It may involve C/EBPβ since several C/EBPβ binding sites are found in the IL-10 promoter [37] and C/EBPβ is a well known down-stream target of IL-6 signaling [41].

### MIP-1α

MIP-1α belongs to the group of CC chemokines that modulate several aspects of the inflammatory response, including trafficking, adhesion and activation of leukocytes, as well as the fever response [42]. Our minimal model identified four regulatory modules for MIP-1α: JNK, p38-PAF, cAMP and STAT1 (Figure 8). In macrophages, MIP-1α mRNA is rapidly induced by TLR ligands and IFNγ (whose effect could be represented by STAT1 in our model), and this effect can be down-regulated by dibutyryl cAMP [43,44]. DNA-binding studies revealed a role for C/EBPβ, NF-κB and c-Ets transcription factors [12]. As discussed earlier, C/EBPβ may be inferred by the presence of JNK in our model. NF-κB may have been omitted due to the high variability of the MIP-1α data leading to a less precise model. Since NF-κB seems to be a false negative predictor and is retained with JNK for all other minimal models, the JNK-NF-κB module is shown activating MIP-1α in Figure 8. MIP-1α mRNA also contains ARE motifs known to be implicated in mRNA stability and translational control [43]. This process is under the control of p38 [45] and, therefore, may be reflected in the p38-PAF component of our model.

### RANTES

RANTES/CCL5 is a CC chemokine that is predominantly chemotactic for monocytes/macrophages and lymphocytes [46]. Three main pathways have been demonstrated to be important for its gene induction in macrophages: JNK, NF-κB and interferon regulatory factors (IRFs) [46]. Transcriptional activation of the RANTES promoter is dependent on specific AP-1 and NF-κB response elements, which are regulated by JNK and NF-κB kinase cascades, respectively [47]. It is well established that IFNγ and TNFα cooperatively induce RANTES gene expression, although no STAT binding elements have been identified in the promoter [48,49]. The synergy between IFNγ and TNFα may involve IRFs since it was demonstrated to require STAT1 activation and to be dependent on protein synthesis [50]. Indeed, IRF-1 was shown to bind the RANTES promoter [51]. As seen previously, LPS, but not the other TLR ligand, activates IRFs via a MyD88-independent pathway [29]. Therefore, the STAT1 and LPS-dependent pathway identified in our minimal model can be explained by the role of IRF-1/IRF-3 (Figure 8).

### TNFα

TNFα is essential for normal host defense in mediating inflammatory and immune responses [52]. Signal transduction mechanisms that regulate TNFα production have been of considerable interest. In macrophages, TNFα production has been shown to undergo transcriptional and post-transcriptional controls [53]. NF-κB is the best described transcriptional activator, with three binding sites on the TNFα promoter [54]. Its inhibition by overexpression of its natural inhibitor IκB alpha reduced LPS-induced TNFα production by 80% [55]. The other transcription factors recruited to the TNFα promoter involve Sp1, the ERK targets Egr-1, Ets and Elk-1 [56], as well as the JNK targets c-Jun and ATF-2 [57]. Transcription of TNFα is augmented by IFNγ [58] and inhibited by the cAMP/PKA pathway [59]. Post-transcriptional regulation of TNFα production also involves ARE elements under the control of p38 [45,60,61]. Therefore, except for the ERK pathway, our minimal model identified the known signaling mechanism responsible for the regulation of TNFα (Figure 8). Moreover, it also identified an independent M-CSF specific pathway. M-CSF treatment was shown to trigger TNFα production by monocytes [62]. However, to our knowledge, the underlying mechanism is not known. This study suggests that it follows a pathway independent of NF-κB, JNK or p38.

Evaluation of our models using literature data shows good agreement, although a precise assessment should be done *in vitro *in RAW264.7 macrophages since regulation of cytokine production is cell-type and sometimes cell-state dependent. Our minimal model covers all known mechanisms of activation of G-CSF and highlights a potential role for p38 in its post-transcriptional regulation. For IL-1α release, besides all known activators, IFNγ and IL-4 are identified as potential novel independent activators. For IL-6 release, four predictors were corroborated by literature data whereas cAMP and IL-4 may be false positives, although the role of IL-4 is controversial. IL-10 response yielded the least convincing model, with a misidentification of NF-κB and a non-identification of p38 and cAMP as positive predictors. Another obvious missing predictor was NF-κB for MIP-1α release. However, in this model, all other important signaling pathways were represented. For RANTES release, all known mechanisms of activation were found. Finally, all known signaling pathways with the exception of ERK were found for TNFα release. This last minimal model also identified a potentially new M-CSF specific pathway for the activation of TNFα. Overall, the performance of our strategy is excellent, with a 1.2% false positive rate and a 13% false negative rate.

## Conclusion

We designed an input-output modeling approach that integrates PCR and exhaustive-search-based model reduction. We have demonstrated that this approach is applicable to heterogeneous types of data through combining western blot phosphorylation and cAMP measurements, and is extendable to other types of data, such as those measured by mass spectrometry. Regarding the issue of scalability to much larger data sets, we note that the PCR part solves a set of linear equations and hence scales well for large systems with thousands of predictors. The minimization part warrants combinatorial optimization, is computationally intensive and hence can go up to exponential complexity in the number of predictors. Nevertheless, it is tractable for up to a few hundred predictors, which is adequate for most cellular intermediate phenotype measurements.

Cytokines mediate pathogenesis of many diseases (for example, chronic inflammatory diseases, autoimmune diseases, cancer). With increasing quantitative knowledge about the important pathways in the production of cytokines, model building as presented in this study will help identify novel targets in order to maximize the efficacy of a drug such that it affects one or few cytokines while minimizing the effect on the homeostasis of other cytokines. The results of the present study demonstrate the power of using heterogeneous cellular data to qualitatively and quantitatively map intermediate cellular phenotypes. These predictive models of the physiological process of cytokine release are important for a quantitative understanding of macrophage activation during the inflammation process.

## Materials and methods

### Data

Single- and double-ligand screen experimental data were obtained from the AfCS Data Center [9]. To generate these data, RAW 264.7 macrophages were stimulated with a variety of receptor-specific ligands applied alone or in combinations of two. Time-dependent changes in signaling-protein phosphorylations, intracellular cAMP concentrations and extracellular cytokines released were measured. Assays included immunoblots to detect phosphorylation of signaling proteins at 1, 3, 10 and 30 minutes after stimulation (AfCS protocols #PP00000177 and #PP00000181 [63]), competitive enzyme-linked immunosorbant assays to measure cAMP concentrations at 20, 40, 90, 300 and 1,200 seconds after stimulation (AfCS protocol #PP00000175 [63]), and a multiplex suspension array system (Bio-Plex, Bio-Rad, #171-F11181) to measure concentrations of cytokines in the extracellular medium at 2 hours, 3 hours and 4 hours after stimulation (AfCS protocols #PP00000209 and #PP00000223 [63]).

### ANOVA analysis

To quantitatively estimate the contributions of various experimental and biological factors to signaling-protein phosphorylations and cytokine release, statistical models of single-ligand screens are defined as:

c_ijk _= μ + T_i _+ L_j _+ E_k _+ TL_ij _+ TE_ik _+ LE_jk _+ e_ijk_

where c_ijk _is the measured response at time T_i _for ligand condition L_j _in experiment E_k_. L is defined as a particular ligand being present or absent (the corresponding control). Interaction term TLK is included in the random error (e). ANOVA were performed on log transformed data (base *e*). Significant terms were identified after correction for multiple testing (Dunn-Sidak method). In the case of protein phosphorylation data, the 30 minutes time point was discarded and the remaining time points (1, 3 and 10 minutes) were each randomly paired to one of the three measurements of basal phosphorylation. Studentized residuals were assessed on residual and quantile-quantile (Q-Q) plots.

### Data pre-processing

The input matrix was constructed from cAMP and signaling-protein phosphorylation data and the output matrix was constructed from cytokine release data. For signaling-protein phosphorylation, a fold change over basal was calculated (AfCS protocol #PP00000181 [63]). For cAMP, the corresponding control concentration was subtracted and one was added. In both cases, the natural logarithm was taken and data were averaged across time points after removing time-series with missing values. Means and standard deviations were obtained from replicate experiments. Most of the measurements had three or more replicates. A few measurements did not have any replicates, but were still incorporated. Extracellular cytokine concentrations were log-transformed after subtraction of the corresponding controls concentration and addition of one. Signal-to-noise ratios were also calculated as the difference between treated and control measurements divided by the standard deviation of the control measurements. Cytokines with an average signal-to-noise ratio lower than five were discarded. The remaining seven cytokines (G-CSF, IL-1a, IL-6, IL-10, MIP-1α, RANTES and TNFα) were retained for further analysis. Time-series with missing values were discarded and outliers, defined as repeats with z-scores outside a 95% confidence interval, were removed. Data were averaged across time points. Means, variances and standard deviations were obtained from replicate experiments. For each cytokine, variance distributions were assessed and stimulation conditions with large variances (outside a 95% confidence interval) were discarded. A matrix of *m *stimulation conditions × *n*_1 _predictors (independent block) was constructed from the mean values (across time-points and repeats) for cAMP and protein phosphorylation measurements. A matrix of *m *stimulation conditions × *n*_2 _responses (dependent block) was constructed from the mean values for cytokines release.

### Identification of significant predictors

Significant predictors (that is, phosphoproteins and ligands) were identified through a PCR [10] and significance-test based procedure. The significant-test was carried out by comparing the predictor coefficients in the PCR model with the standard deviation in the coefficients corresponding to a PCR model with random outputs. The predictors with a ratio higher than a threshold, *r*_*th *_= 1.96 corresponding to 95% confidence, were considered significant. In principal, the methodology is similar to the bootstrap method in which randomly shuffled outputs are used to develop random models [11], but in our novel procedure these random models are never actually identified. Instead, an indirect procedure is used in which the desired standard deviation is calculated implicitly by utilizing the latent variables of the input data and the standard deviation of the population of output data. The procedure is given below.

#### Step 1: Principal component decomposition of the input data

Let *X *be the normalized input data (zero-mean, unit-standard deviation), of size *m *× *n*_1 _and *Y *be the normalized output data (zero-mean), of size *m *× *n*_2_. Compute the eigen values (λ_*i*_, *i *= 1,..., *n*_1_) and eigen vectors (loadings, *v*_*i*_) of the covariance matrix of *X*, *S*. Calculate the scores (latent variables, *T*_*i*_): *T*_*i *_= X *v_*i*_.

#### Step 2: PCR model

For *k *latent variables, let *V *= [*v*_1 _*v*_2 _... *v*_*k*_], Λ_*k *_= *diag*([λ_1 _λ_2 _... λ_*k*_]), and *T *= [*T*_1 _*T*_2 _... *T*_*k*_] = X*V. Then, the PCR model for j^th ^output (*Y*_*j*_) is:

*Y_j _*= X*B_j,k_

with the coefficients

*B_j,k _*= V*(Λ_k _*(*m *- 1))^-1^*T^T ^*Y_j_

#### Step 3: Ratio of the coefficients *B_j,k _*to the standard deviation of coefficients for random models (σ_*j,k*_)

In a boot-strap approach, many random shufflings of the output are considered. For each, a model is built. Then the standard deviation (σ_*j,k*_) of the coefficients in these models is calculated. Here we use a novel implicit (indirect) approach to estimate σ_*j,k*_. Consider a random model with coefficients  corresponding to the output values , the *l*^th ^random shuffling of the j^th ^output *Y_j_*. Then:

 = V * (Λ_k _* (*m *- 1))^-1 ^* T^T ^* 

and hence

σ_*j,k *_= *std*() = *diag*(V * (Λ_k _* (*m *- 1))^-1 ^* *V^T^*)* *std*()

where *std *refers to standard deviation and diag (*A*), *A *being a square matrix, is a column vector containing the diagonal elements of *A*. Since (∀ *l*) belong to the same population as *Y_j_*, *std*() ≈ *std*(*Y_j_*) (observed computationally too), and hence:

σ_*j,k *_= *std*() ≈ *diag*(V * (Λ_k _* (*m *- 1))^-1 ^* *V^T^*)* *std*(*Y_j_*)·*r_j,k _*= *B_j,k_*/σ_*j,k*_.

#### Step 5: Identification of significant predictors

Repeat Steps 2 and 3 for *k *= *k*_min _,..., *k*_max_, where *k*_min _and *k*_max _are the number of latent variables needed to capture 80% and 95%, respectively, variance in *X*. Compute the average of *r*_*j*,*k*_, , and the threshold *r*_*th *_= the confidence interval of normal distribution for a specified significance (*r*_*th *_= 1.96 for 95% confidence, *t* test with infinite degree of freedom). The *i*^th ^predictor is significant if  >*r_th _*( is the *i*^th ^element of ).

#### Step 6: Development of a model based upon PCR

Choose the number of latent variables (*k*) corresponding to the minimum fit-error to develop a model.

One model is developed for each cytokine (output). First, all the measured phosphoproteins and cAMP are used to develop a phosphoproteins model (PP-model) to explain extracellular cytokine levels from signaling pathway activation. Then, the residuals are calculated and used to identify if the inclusion of one or more ligands in the model can significantly improve the fit of the data. If so, it is inferred that the PP-model alone does not capture all the important pathways and that the inclusion of ligands captures pathways from the ligands to the output through unmeasured signaling-proteins (Figure 1). Here ligands serve as predictors and residuals serve as outputs. In the residuals-model, *r*_*th *_=  * 1.96 = 2.7719 is used since residuals themselves have a strong random component. The factor  corresponds to the standard deviation of difference of two random variables (that is, mean of random coefficients – random coefficients) drawn from standard normal distribution.

### Development of minimal models

To reduce the number of false-positives, a model with a minimal number of predictors (minimal model) is developed that has a statistically similar fit-error as the detailed model with all the predictors. A two-level procedure is used. At level one, using the significant phosphoproteins identified based upon the detailed model, one or more minimal PP-models are developed by a combined sequential and combinatorial (exhaustive search) model-reduction procedure. Once a minimal PP-model is generated, the residuals are generated for this minimal PP-model. At level two, the residuals are used to identify important ligands by developing a minimal residuals model using the same approach. The overall minimal model is the combination of the minimal PP-model and the minimal residuals model. The procedure for the identification of the minimal model containing the necessary and sufficient set of predictors is summarized below. This procedure is used at both level one and level two for each cytokine.

Starting with a model that includes all the significant predictors, to test if the model is good, the following criteria are used:

1. Statistically same fit-error for the minimal models and the detailed model (F-test): let *e*_*d *_and *e*_*r *_be the root-mean-squared-errors (RMSE) for the detailed and the candidate minimal model. This criterion is satisfied (that is, null hypothesis H_0 _is accepted) if / <*finv*(*p*, *d*_1_, *d*_2_) where *p *= 1 - α, α is the significance-level (0.05), and *d*_1 _and *d*_2 _are the degrees of freedom for  and , respectively. For the residuals model, instead of *e*_*d*_, the fit-error for the significant-predictors model (*e*_s_) is used to avoid over-fitting.

2. The fit-error for minimal models should be statistically lesser (F-test used) than the fit-error for a zero-predictor model (mean-model), that is, the alternative hypothesis (H_1_) is accepted. Else, the mean-model is the minimal model. The logic behind this criterion is that if a model with one or more predictors does not improve the fit over a trivial model, then those predictors should not be included in the minimal model. For this test, *p *= 0.95 is used for the PP-model and *p *= 0.68 (that is, somewhat lesser improvements also are accepted) is used for the residuals model.

If the model satisfies the two criteria listed above, eliminate the least significant predictor from the current list of predictors (based upon the original ranking from the detailed model). Develop a model using the remaining predictors and test if the model satisfies the two criteria. Repeat until no further reduction is possible. If this minimal model has more than one predictor then test all possible combinations of one or more predictors (from the original list of all significant predictors). During this phase, it is also required that the signs of the coefficients of the predictors in the minimal model be the same as the sign of the coefficients of the corresponding predictors in the detailed model. The smallest good model(s) are the minimal model(s). If multiple minimal models are generated, then the model with least fit-error is considered.

To validate the minimal models, test data are used. If validation fails, the test data are also included in the training set and the model-reduction procedure is repeated. Additional details are provided in Additional data file 1.

Matlab code and the data can be obtained upon request.

## Additional data files

The following additional data are available with the online version of this paper. Additional data file 1 contains a detailed description of the procedure for the validation of the model.

## Supplementary Material

Additional File 1 A detailed description of the procedure for the validation of the model. Click here for file

## Figures and Tables

**Table 1:** Predictors identified in the PCR minimal model

Cytokine	Signaling pathways	Ligands
G-CSF	JNK lg	PAF (-)
	NF-κB p65	
	p38	
IL-1α	JNK lg	IFNγ
	NF-κB p65	IL-4
IL-6	cAMP (-)	IFNβ
	JNK lg	IL-4
	NF-κB p65	LPS
IL-10	JNK sh	IL-4
	NF-κB p65	IL-6
MIP-1α	cAMP (-)	PAF (-)
	JNK lg	
	p38	
	STAT1α	
RANTES	JNK lg	LPS
	NF-κB p65	
	STAT1β	
TNFα	cAMP(-)	IFNγ
	JNK lg	M-CSF
	NF-κB p65	PAF (-)
	p38	

(-), negative predictor.

**Table 2:** Cytokine gene regulation

Cytokine	Signaling pathways/transcription factors
G-CSF	NF-κB, C/EBPβ, Oct, post-transcriptional regulation
IL-1α	NF-κB, AP-1, Sp1
IL-6	NF-κB, AP-1, Sp1, IRF-1, C/EBPβ
IL-10	C/EBPβ, C/EBPδ, Sp1, cAMP/PKA
MIP-1α	NF-κB, Ets, C/EBPβ, cAMP/PKA, posttranscriptional regulation
RANTES	NF-κB, AP-1, IRF-1, IRF-3
TNFα	Egr-1, Ets/Elk, NF-κB, c-jun/ATF-2, cAMP/PKA, post-transcriptional regulation (p38 dependent)

References can be found in the text.
