# Supplementary material for: Identification of signaling components required for the prediction of cytokine release in RAW 264.7 macrophages
Source: Genome Biol. 2006 Feb 20;7(2):R11. doi: 10.1186/gb-2006-7-2-r11 (PMC1431720; doi:10.1186/gb-2006-7-2-r11)
Supplement: Additional File 1 — A detailed description of the procedure for the validation of the model. [file gb-2006-7-2-r11-S1.doc]

**Supplementary Material To**

# Identification of signaling components required for the prediction of cytokine release in RAW 264.7 macrophages

Sylvain Pradervand, Mano R. Maurya and Shankar Subramaniam

**Validation of the model**

The procedure for modeling with validation is given below. Dataset 1 and dataset 2 refer to training and validation data (see also the subsection ‘Validation of the model’ in the ‘Materials and methods’ section of the main manuscript).

1. Use dataset 1 for training (modeling).
2. Level-1: For a chosen output (cytokine), develop a minimal PP-model (level-1). Test the model against dataset 2.
3. If successful, calculate residuals and go to Step 4, else include dataset 2 (if already not included) for training and Go to Step 2.

If the inclusion of dataset 2 for training (a) also does not validate dataset 2, or (b) it validates, but model based upon dataset 1 also validated and was of smaller size, or (c) it does not validate dataset 2 and the number of predictors is reduced to zero then force that only dataset 1 be used for training. Go to Step 2. In this case, the minimal PP-model based upon dataset 1 is accepted even though it may not validate dataset 2. Go to Step 4.

1. Use the residuals for dataset 1 for training.
2. Level-2: Develop a minimal residuals-model. Test it against the residuals for dataset 2.
3. If successful, stop else go to Step 7.
4. If at level-1 dataset 2 is not already included or exclusion of dataset 2 from training is not forced, include dataset 2 for training at level-1. Go to Step 2. Else include the residual for dataset 2 at this level (level-2) for training, go to Step 5.

If the inclusion of residuals for dataset 2 for training also does not validate dataset 2 *and* the number of predictors is reduced to zero then force that only the residuals for dataset 1 be used for training. Go to Step 5. In this case, the minimal residuals-model based upon residuals for dataset 1 is accepted even though it may not validate the residuals for dataset 2. Go to Step 4.
